# The Reason Why

**Published:** 1891-04

**Authors:** Samuel Swan

**Affiliations:** New York


### THE REASON WHY.

Samuel Swan, M. D., New York.

Hahnemann remarks in *Lesser Writings*, p. 502: "We observe a few diseases that always arise from *one and the same cause*, namely, the miasmatic maladies: hydrophobia, the venereal disease, the plague of the Levant, yellow fever, small-pox, cowpox, the measles, and some others, which bear upon them the distinctive mark of always remaining diseases of a *peculiar character;* and because they arise from a contagious principle that always remains the same, they also always retain the same character, and pursue the same course, excepting as regards some accidental concomitant circumstances, which, however, do not alter their essential character.

"Probably some other diseases, which we cannot show to depend on a peculiar miasm, as gout, marsh-ague, and several other diseases that occur here and there endemically, besides a few others, also arise either from a single unvarying cause or from the confluence of several definite causes that are liable to be associated and that are *always the same*, otherwise they would not produce diseases of such a specific kind, and would not occur so frequently.

"These few diseases, at all events those first mentioned (the miasmatic), we may therefore term *specific*, and when necessary bestow on them *distinctive appellations.*

"If a remedy has been discovered for one of these, *it will always be able to cure it*, for such a disease always remains essentially identical, both in its manifestations (the representatives of its internal nature) and in its cause."

I have thus far quoted Hahnemann's words concerning the specific or fixed diseases, and add to his list a few of the "some others" which he mentions. These are fixed diseases, always diagnosed by their unvarying character; diphtheria, scarlet fever, typhus fever, eczema, erysipelas, itch, septicæmia, scirrhous, cancer, lupus, leprosy, glandular diseases, gonorrhœal rheumatism, and tuberculosis.

Repeated experiments by myself and other physicians with the poison of these specific diseases, obtained from the morbose products of such diseases, have proved that such poisons *potentized*, will invariably cure the disease from which they were obtained, *except* when some other miasm is present and obstructs the curative action, notably psora.

Hahnemann also says, "All the other innumerable diseases exhibit such a difference in their phenomena that we may safely assert that they arise from a combination of several dissimilar causes." These diseases, which would more properly be termed sicknesses, are so different that each one of them occurs scarcely more than once; never occurring before or since in the same manner, there never can be found a specific remedy for them, and as Hahnemann says "they require no names—we are only required to cure them."

Hahnemann has evidently used these morbose poisons, for he says, in *Chronic Diseases*, vol. I, p. 195, "In the subsequent list of antipsoric remedies, no isopathic remedies are mentioned." The reason he gives is "that their effects upon the *healthy* organism have not yet been sufficiently ascertained." It would seem from this that *he had these isopathic remedies, had potentized them, had used them on the sick, had found how valuable they were, had partially proved them in healthy organisms*, but not so *thoroughly* as to warrant his giving them to the profession.

He thus disposes of Isopathy. On page 196, *Chronic Diseases*, he says: "*I call Psorin a homœopathic antipsoric, because if the preparation*" (potentization) "*of Psorin did not alter its nature to that of a homœopathic remedy, it never could have any effect upon an organism tainted with that identical virus.*"

The corollary is inevitable. The potentization of the *isopathic* product *makes it homœopathic to the disease which produced it*, and it cannot have any curative effect on that disease *till* potentized, but *when* potentized it does have an effect, and the effect must be homœopathic, and, therefore, of necessity a curative effect, or, in other words, "*Morbose poison will cure the disease which produced it, if given in a high potency.*" *Had not Hahnemann tried morbose products empirically on those sick of the diseases which had produced those products, he would not have said that unless these were so altered by potentization they never could "have any effect on an organism tainted with that same identical virus.*"

Hahnemann did not make public any remedy, no matter how much *he* knew about it, till it had been proved according to the rule laid down, but in the same volume he gives some toxical symptoms of psora, syphilis, and sycosis which were probably the key-notes from which he prescribed for those "tainted with that same identical virus." He evidently believed that later the problem of the use of these morbose poisons would be solved, as he says, in the foot-note of paragraph 56, page 194, of the *Organon*, "but supposing this were possible, and it would deserve the name of a valuable discovery," etc. The problem *is* solved by the use of those poisons in the *high potencies*.

The *numerous symptoms* that appear when a person is attacked by any of these fixed diseases are a sufficient proving of the poison for the *cure of the disease;* a proving on healthy persons would not add to the curability of the remedy, it would only give a variety of symptoms whereby the poison could be diagnosed in diseases, where there was no other indication of its presence. An aggravation at night, ceasing with daylight, is always indicative of syphilism. If any one objects to the absence of a proving, it is his duty to make the proving himself. If I am in error, I am willing to acknowledge it when shown to me—but the unfailing success attendant on this mode of treating fixed diseases is of itself proof of its truth.

February, 1891.

